# Retrieval Intention Modulates the Effects of Directed Forgetting Instructions on Recollection

**DOI:** 10.1371/journal.pone.0104701

**Published:** 2014-08-20

**Authors:** Xin Xiao, Heather D. Lucas, Ken A. Paller, Jin-hong Ding, Chun-yan Guo

**Affiliations:** 1 Beijing Key Laboratory of Learning and Cognition, Department of Psychology, Capital Normal University, Beijing, China; 2 Beckman Institute, University of Illinois at Urbana-Champaign, Urbana, Illinois, United States of America; 3 Department of Psychology, Northwestern University, Evanston, Illinois, United States of America; University of Groningen, Netherlands

## Abstract

The neurocognitive basis of memory retrieval is often examined by investigating brain potential *old/new effects*, which are differences in brain activity between successfully remembered repeated stimuli and correctly rejected new stimuli in a recognition test. In this study, we combined analyses of old/new effects for words with an item-method directed-forgetting manipulation in order to isolate differences between the retrieval processes elicited by words that participants were initially instructed to commit to memory and those that participants were initially instructed to forget. We compared old/new effects elicited by to-be-forgotten (TBF) words with those elicited by to-be-remembered (TBR) words in both an explicit-memory test (a recognition test) and an implicit-memory test (a lexical-decision test). Behavioral results showed clear directed forgetting effects in the recognition test, but not in the lexical decision test. Mirroring the behavioral findings, analyses of brain potentials showed evidence of directed forgetting only in the recognition test. In this test, potentials from 450–650 ms (P600 old/new effects) were more positive for TBR relative to TBF words. By contrast, P600 effects evident during the lexical-decision test did not differ in magnitude between TBR and TBF items. When taken in the context of prior studies that have linked similar parietal old/new effects to the recollection of episodic information, these data suggest that directed-forgetting effects manifest primarily in greater episodic retrieval by TBR than TBF items, and that retrieval intention may be important for these directed-forgetting effects to occur.

## Introduction

A great deal of research interest has been focused on directed forgetting (DF), or the process of intentionally forgetting information that has been learned. In experiments that examine DF, participants are typically instructed to commit to memory only a subset of the stimuli (words, faces, etc.) with which they are presented, and to refrain from memorizing other stimuli presented in the same experiment. When participants’ memory is tested later, it is typically found that stimuli that were initially marked as to-be-remembered (TBR) are indeed recognized or recalled with greater accuracy relative to items that were marked as to-be-forgotten (TBF). These findings have been obtained when TBR and TBF cues are intermixed within the same study list (referred to as *item method* directed forgetting), as well as when entire study lists are presented and then subsequently designated as TBR or TBF (referred to as *list method* directed forgetting). Together, this body of research indicates that people have some ability to suppress irrelevant or unwanted information from memory.

Research thus far has focused primarily on DF effects on *intentional retrieval*, or the ability to deliberately retrieve previously-studied information. However, remembering can also occur incidentally, without retrieval intention. Indeed, it seems likely that unwanted memories are particularly apt to be retrieved in a manner that is incidental rather than intentional. The repeated, involuntary recollection of negative past events constitutes a key symptom of post-traumatic stress disorder (PTSD; for review see [1]), and similar memory intrusions characterize disorders such as depression [2] and certain anxiety disorders [3]. Moreover, it has been argued that the involuntary retrieval of both negative and non-negative information from memory is a pervasive but understudied aspect of human experience (for reviews, see [4], [5]). For these reasons, it is important to investigate the potential for DF instructions to affect incidental as well as intentional expressions of memory.

At first blush, it might seem that any effects of DF instructions on later memory would be magnified when retrieval is incidental relative to when it is intentional. Intentional retrieval typically involves an effortful search of memory for the to-be-retrieved information, as mirrored by the greater recruitment of right prefrontal regions during intentional memory tasks (e.g., when an individual is in “retrieval mode”) relative to when similar tasks are completed without retrieval intention (e.g., [6], [7]). It is plausible that this intentional search process can aid in the recovery of weaker memory traces associated with TBF stimuli in addition to the typically stronger traces associated with TBR stimuli. Incidental retrieval, by contrast, may tend to be limited to those memories that are most accessible (e.g., those associated with TBR items).

Nonetheless, there may also be reason to suspect that adopting an intentional retrieval mode can disproportionately facilitate the recovery of memories for TBR items. Although theories of directed forgetting differ (e.g., [8]–[12]), a point of general agreement is that TBR items are subject to deeper or more elaborative encoding relative to TBF items. According to the encoding specificity hypothesis [13], the memorial benefits of elaborative processing during encoding will be most pronounced when participants engage in the same types of processing at the time of retrieval. Jacoby (1984) has argued that individuals who are attempting intentional retrieval are likely to do just that, elaborating retrieval cues in the same manner as they did during encoding [14]. Consistent with this idea, it is well known that a change in environmental context between study and test has a negative effect on memory. However, it has been shown that this effect can be diminished by instructing participants at the time of testing to imagine that they are in the study context, thereby encouraging them to mentally reinstate their encoding strategies [15], [16]. These types of retrieval strategies are, by definition, not engaged when retrieval is incidental. Thus, the beneficial effects of elaborative encoding of TBR cues may be less effective in aiding incidental relative to intentional retrieval.

To our knowledge, no prior studies have investigated DF effects on incidental or involuntary explicit memory retrieval so as to distinguish between these possibilities. Although some studies have administered implicit memory tests following DF instructions, they have done so with the goal of examining DF effects on implicit or nonconscious expressions of memory such as priming. Priming refers to the facilitated processing of stimuli as a result of prior experience, and can occur without conscious awareness of repetition. Findings thus far have been mixed as to whether DF instructions can reliably influence priming, with some studies finding effects [10], [17], [18] and others not [19]–[22]. However, effects of DF instructions on priming do not necessarily imply parallel effects of DF instructions on incidental recollection. While the relationship between priming and incidental recollection has been a topic of some controversy (e.g., [23]), there is evidence that these forms of memory have different neural correlates [7] and are differentially affected by certain experimental manipulations [24], [25]. Thus, it is likely that DF effects on priming tasks are at least partially independent of any DF effects that occur for incidental recollection.

The study of incidental explicit memory is inherently challenging due to the inability of the experimenter to directly interrogate participants’ retrieval experiences. Because participants arguably cannot report on retrieval success without adopting some type of intentional retrieval mode, it can be problematic to attempt to obtain direct reports of incidental retrieval. However, event-related potentials (ERPs) have shown promise as an indirect means to query explicit memory. In particular, ERP old/new effects – or the difference in neural activity when correctly recognized repeated items are compared to new items – have demonstrated sensitivity to similar retrieval outcomes during both intentional and incidental tests of memory [24], [26]. The present study thus uses ERPs in conjunction with an item-method directed forgetting paradigm to gain traction on the relationship between retrieval intention and the efficacy of directed forgetting- that is, whether incidental explicit memory retrieval is affected by DF instructions in a manner similar to intentional explicit memory retrieval.

ERPs have been used extensively in prior research to examine old/new effects on both explicit and implicit memory tests. When words or meaningful images are used as stimuli, repeated items tend to elicit more positive brain potentials relative to unstudied items around ∼400 ms, consistent with an effect on N400 potentials. N400 potentials have been shown to reflect facilitated semantic or lexical access for repeated words [27], which is believed to underlie certain types of priming in implicit memory tests. Thus, while these ERPs may co-occur with or contribute to certain explicit memory decisions, it is believed that they do not directly reflect explicit memory *per se* [28]–[30]. Of most relevance to the present research, previously-studied items also typically elicit more positive P600 potentials relative to unstudied items. These so-called late parietal old/new effects have been widely attributed to episodic retrieval processes, and particularly to *recollection*, which is characterized by the retrieval of contextual details about a prior encounter with the stimulus [29]–[33]. Unlike N400 potentials, P600 potentials have been convincingly dissociated from priming effects even when they occur in the context of indirect or implicit memory tasks. In Paller, Kutas, and McIssac (1995), for example, it was found that P600 potentials recorded during a lexical decision test varied systematically with experimental manipulations – such as a depth-of-encoding manipulation – that affect the likelihood of incidental recollection without affecting priming [24]. In a similar vein, the present study will directly compare P600 old/new effects for TBR words with those for TBF words in the context of both an explicit memory test (recognition test) and an implicit memory test (lexical decision test) so as to provide insight into the potential for directed forgetting instructions to influence incidental recollection.

A small number of ERP studies have examined old/new effects in the context of directed forgetting paradigms. However, no study thus far has been able to directly compare P600 old/new effects for TBR and TBF words across both explicit and implicit memory tests. In Ullsperger, Mecklinger, and Muller [34], TBR items were associated with both early frontal and late parietal old/new effects in a recognition test, but only the earlier effect was evident for TBF items. In Paz-Caballero and Menor [21], late parietal old/new effects were present for both TBR and TBF items on a recognition test, though these effects were larger for TBR items. However, ERP old/new effects were entirely absent for both TBR and TBF words in this study on an implicit categorization task that was administered to a separate group of participants. Similar findings were obtained in a recent study of directed forgetting that administered a lexical decision test followed by a recognition memory test to each participant [22]. As in Ullsperger et al. [34], both TBR and TBF items in the recognition test were associated with early old/new effects, whereas only TBR items evinced P600 old/new effects. However, no old/new effects were found on the implicit memory test for either category of words, similar to the findings of Paz-Caballero and Menor [21].

We reasoned that P600 old/new effects may have been obscured in the implicit memory tests of Paz-Caballero et al. [21] and Van Hooff et al. [22] because participants were exposed to a very large number of words in the study phases in these experiments (100 and 120 words per block, respectively), potentially leading to both high levels of interference and rather weak memory traces for studied words on the incidental memory tests. Thus, to raise the probability of simultaneously obtaining both directed forgetting and old/new effects in our experiment, we limited the number of studied words to 40 per block. Given this smaller number of stimuli, we expected that P600 old/new effects would be evident for both TBR and TBF items in both the recognition test and the lexical decision test. Analyses of ERPs at test will allow us to examine whether or not incidental recollection occurs during lexical decision tests to a greater extent for TBR relative to TBF words, and whether these DF effects (if present) differ in magnitude when compared to those observed during the recognition memory test.

Finally, as previously mentioned, a related literature deals with whether or not DF instructions at study can influence implicit memory phenomena such as priming. The potential for DF to influence priming is of interest because it can speak to current theoretical controversies about the mechanisms of directed forgetting. Specifically, it has been suggested that pure “selective encoding” accounts of directed forgetting would be unable to account for DF effects on certain forms of implicit memory, including lexical decision priming [10]. Because priming on the lexical decision task has been shown to be insensitive to encoding manipulations (such as depth of study [24]), it has been argued that additional *active inhibition* of TBF stimuli would be necessary to produce differential priming effects on TBR and TBF items. Interestingly, although DF effects on priming have been absent in all ERP studies thus far, such effects have sometimes been found in behavioral studies, particularly in experiments with shorter study-test blocks such as this one [10], [18], [35]. Thus, in addition to our primary ERP analyses, behavioral analyses of DF effects on priming can provide an additional contribution to our understanding of directed forgetting and its underlying mechanisms.

## Methods

### Subjects

Fourteen right-handed students (7 females) from different universities, ranging in age from 18 to 23 years (mean = 19.4) participated in the experiment. All participants were native Chinese speakers and had normal or corrected-to-normal vision. Each participant gave written informed consent and received monetary compensation. This research was approved by the Human Research Ethics Committee at Capital Normal University.

### Stimuli

The materials consisted of 720 two-character low frequency Chinese nouns (below 30 occurrences/million, Modern Chinese Frequency Dictionary, Beijing Language College, 1986), as well as 480 two-character non-words. The non-words were constructed by combining the first characters of real two-character words with the second characters of other real two-character words. Ratings from 30 individuals who did not participate in the present study confirmed that none of the resulting two-character combinations formed pre-existing words. The words and non-words were then randomly divided into six lists, forming six blocks. In each block, a list consisting of 20 TBR and 20 TBF words was used in the study phase, and a list of 40 studied words, 40 new words, and 80 non-words was used in the lexical decision task. Finally, a list containing 40 studied words and 40 new words was employed in the recognition task. Words that were presented for the first time in a lexical decision task were not included in the recognition tasks. In each block, the stimuli were presented in a pseudo-random order, with the constraint that no more than three items from the same stimulus category (e.g., TBF or TBR in the study phase, word or pseudoword in the lexical decision phase, and old or new in the recognition test phase) were permitted to appear consecutively.

### Procedure

**Figure 1:**
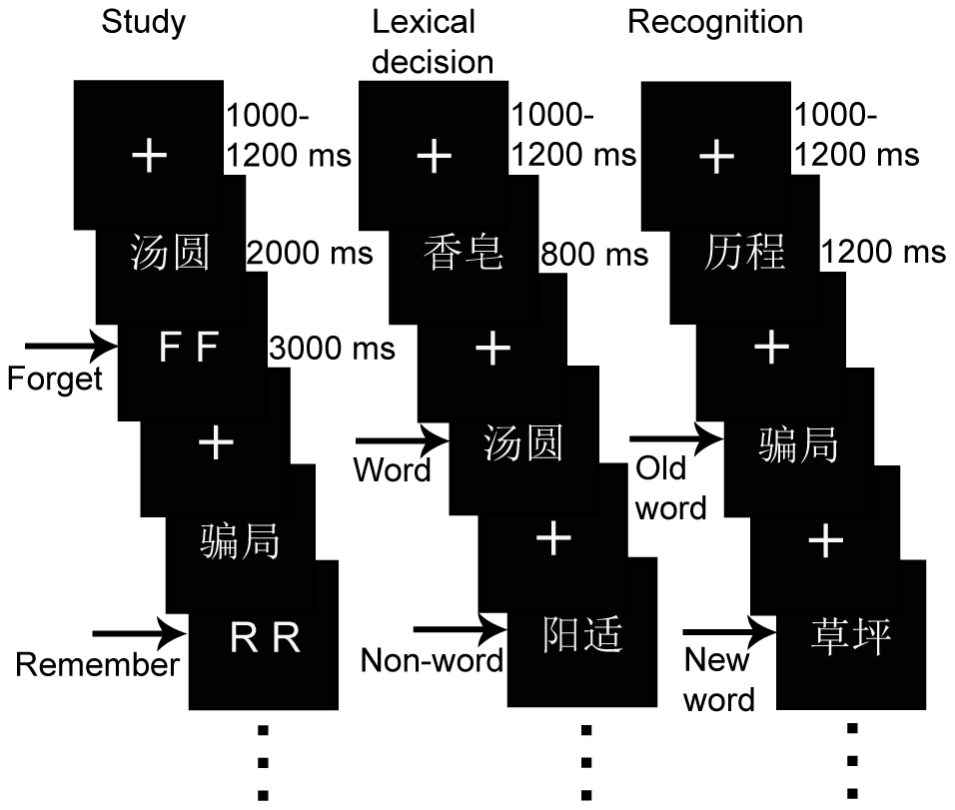
Experimental Procedure. The experiment consisted of six study-test blocks. Each block consisted of a study phase (left pane), which was followed first by a lexical decision test (center), and then by a recognition test (right pane).

Stimuli were presented at the center of the screen and subtended 4.28°×2.26° of the visual angle. The experiment was divided into six blocks. Each block consisted of three consecutive tasks: a study phase, a lexical decision test, and a recognition test (see Figure 1).

Prior to each study phase, subjects were told that each item would be followed by either a “remember” cue (RR) or a “forget” cue (FF), and were instructed to attempt to remember the RR items and to forget the FF items. Each trial began with the presentation of a fixation cross that was displayed with an ISI ranging from 1000 to 1200 ms, followed by a word that was displayed for 2000 ms. Each word was followed by either an RR or FF cue, which was displayed for 3000 ms. After each study phase, participants completed a 30 s distracter task during which they counted down from 3 digit numbers (such as 572, 872, or 378).

During the lexical decision tasks, each trial began with the presentation of a fixation cross for 1000–1200 ms, followed by the presentation of a stimulus for 800 ms. The subjects were asked to press one of two buttons indicating whether the stimulus was a word or a non-word. Both speed and accuracy were emphasized.

During the recognition tasks, trials began with the presentation of a fixation cross for 1000–1200 ms, followed by the presentation of a stimulus for 1200 ms. Subjects were required to press one of two buttons to indicate whether the word was old or new.

### Recording of ERPs

ERPs were extracted from scalp electroencephalographic recordings from 62 scalp channels using Ag/AgCl electrodes embedded in an elastic cap at locations corresponding to the International 10–20 System. Voltage was referenced to a left mastoid electrode online and re-referenced to the average of the right and left mastoid signals during off-line analysis. The ground electrode was placed between electrodes Fpz and Fz. The electrooculogram was recorded from electrodes that were placed above and below the left eye as well as on each outer canthus. Signals were recorded with a band pass of 0.05–100 Hz, and sampled at a rate of 500 Hz. Electrode impedance was kept below 5 kΩ. During offline analysis, EEG signals were filtered with a band pass of 0.05–40 Hz. Trials containing ocular and movement artifacts with amplitudes that exceeded a range of ±75 µV were excluded from analysis. Each 1100 ms averaging epoch began 100 ms prior to stimulus onset. Mean pre-stimulus amplitudes were subtracted to correct for baseline variability. Statistical comparisons were performed using repeated-measures ANOVAs (criterion *p* = 0.05) with Greenhouse-Geisser correction for non-sphericity where appropriate.

## Results

### Behavioral data

The mean accuracy and reaction times for TBR, TBF, and new items are shown in Table 1 for both the implicit and the explicit memory tests. For both test types, the effects of old/new status and directed-forgetting instructions on participants’ response accuracy and reaction times were assessed via one-way, repeated measures ANOVAs with the factor of stimulus category (TBR/TBF/New). Significant main effects were further analyzed via two-tailed, paired t-tests.

**Table 1:** Mean response accuracy (RA) and reaction time (RT) for each condition.

Conditions	Explicit test		Implicit test	
	Mean RA in % (*SE*)	Mean RT in ms (*SE*)	Mean RA in % (*SE*)	Mean RT in ms (*SE*)
TBR	90.5 (1.9)	642 (14.7)	97.4 (0.6)	576 (9.1)
TBF	84.6 (2.5)	659 (17.5)	96.6 (0.7)	570 (11.2)
New	91.5 (1.4)	687 (16.5)	89.6 (1.1)	604 (10.5)

#### Behavior – Explicit Memory Test

To assess the effects of the directed forgetting cues on participants’ recognition accuracy, statistical analyses focused on the percentage of “old” responses registered for each stimulus category on the explicit memory test. The main effect of stimulus category was significant [*F*(2,26) = 10.69, *p*<0.001]. Follow-up comparisons revealed that both the hit rate for TBR words and the hit rate for TBF words were significantly greater than the false alarm rate for new words [*t*(13) = 33.39, *p*<0.001 for TBR words; *t*(13) = 26.68, *p*<0.001 for TBF words], indicating above-chance memory for both stimulus categories. Furthermore, significantly more hits were registered for TBR relative to TBF words [*t*(13) = 3.68, *p*<0.01], indicating that the directed forgetting manipulation affected performance on the explicit memory test.

These accuracy effects were mirrored in participants’ reaction times on the explicit memory test. As in the analysis of recognition judgments, a main effect of stimulus category emerged [*F*(2,26) = 10.69, *p*<0.001]. Paired comparisons revealed that reaction times were significantly shorter for both TBR words compared to new words [*t*(13) = 4.28, *p*<0.001] and for TBF words compared to new words [*t*(13) = 2.31, *p*<0.05]. Moreover, reaction times were also significantly shorter for TBR relative to TBF words [*t*(13) = 2.93, *p*<0.05].

#### Behavior - Implicit Memory Test

On the implicit memory test, a main effect of stimulus category emerged for the analysis of response accuracy [*F*(2,26) = 67.70, *p*<0.001]. Pairwise t-tests revealed that accuracy was significantly higher for TBR words relative to new words [*t*(13) = 8.17, *p*<0.001], as well as for TBF words relative to new words [*t*(13) = 10.33, *p*<0.001]. However, accuracy did not differ between TBR and TBF words [*t*(13) = 1.74, *p = *0.105]. Note that the ability to interpret the nonsignificant difference in accuracy is complicated by the presence of a ceiling effect, as indicated by the very high levels of accuracy for both TBR words (97.4%) and TBF words (96.6%) on the implicit test.

A significant main effect of stimulus category also emerged for the analysis of participants’ reaction times [*F*(2,26) = 43.96, *p*<0.001]. As with analyses of accuracy, pairwise t-tests revealed that reaction times were significantly faster for both TBR words compared to new words [*t*(13) = 7.56, *p*<0.001], and for TBF words compared to new words [*t*(13) = 8.26, *p*<0.001]. However, no significant difference was present between reaction times to TBR and TBF words [*t*(13) = 1.75, *p = *0.104].

To summarize, analyses of both reaction times and accuracy suggest that the directed forgetting manipulation affected performance on the explicit memory test, but not on the implicit memory test.

### ERP data

Analyses of ERPs recorded during the test phases focused on mean amplitudes over the latency intervals of 350–450 ms and 450–650 ms, which were selected to correspond to ERP effects in the N400 and P600 range, respectively. Old/new effects in the N400 range typically present with a fronto-central distribution, whereas P600 old/new effects tend to be centro-parietally distributed (e.g., [29], [30]). Accordingly, similar statistical analyses for the two test types took the form of a 3 (Stimulus Category: TBR/TBF/New) x 3 (Electrode: Fpz/Fz/Cz) ANOVA for the 350–450 ms interval, and a 3 (Stimulus Category: TBR/TBF/New) x 3 (Electrode: Cz/Pz/Oz) ANOVA for the 450–650 ms interval. In addition, comparisons of ERPs elicited by TBR and TBF items at study are presented as Supporting Information (Figure S1). These study-time analyses confirmed that TBR items were encoded differently from TBF items (Text S1).

#### ERPs – Explicit memory test

**Figure 2:**
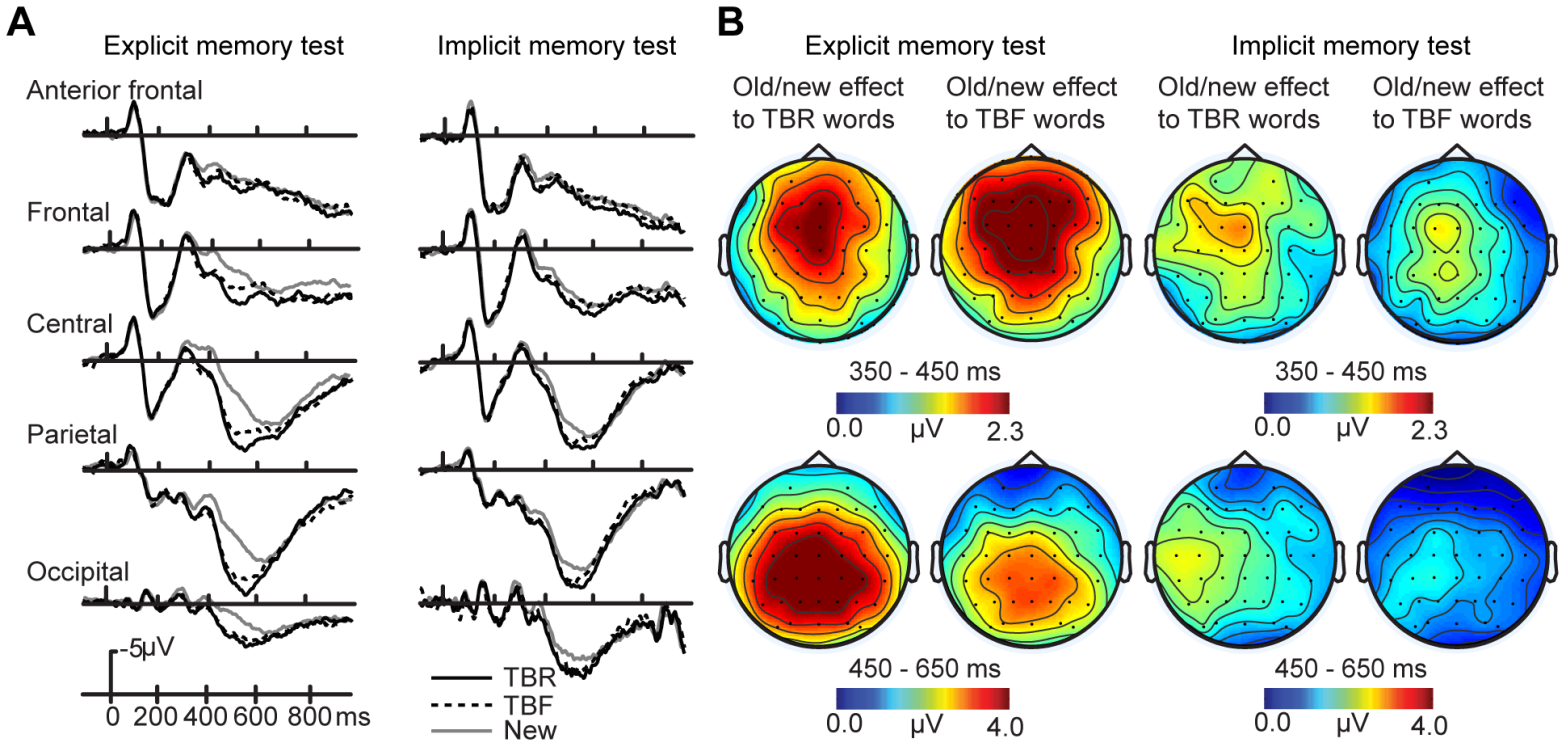
ERP differences among TBR, TBF, and new words on the implicit and explicit memory tests. A) Waveforms plotted at five midline electrodes. Significant differences between TBR and TBF words occurred over the 450–650 ms window on the explicit memory test only. B) Topographical plots depict ERP old/new effects for both TBR items and TBF items in the explicit and implicit memory tests from 350–450 ms (upper plot) and 450–650 ms (lower plot).

Figures 2a and 2b (left panels) show that approximately 350 ms after test word onset, positive old/new effects with a frontocentral distribution were visible for both TBR and TBF words compared to new words. These anterior differences encompassed the 350–450 ms range chosen to represent N400 old/new effects. However, these ERPs did not appear to differ between TBR and TBF words. Later positive old/new effects with centoparietal distributions (450–650 ms, P600 old/new effects) were also apparent for both TBR and TBF words compared to new words. Furthermore, ERPs in this time window appeared to be more positive for TBR relative to TBF words. These observations were substantiated by formal statistical analyses over each latency window.

Over the 350–450 ms interval, a significant main effect of stimulus category emerged [*F*(2,26) = 54.22, *p*<0.001], as well as a marginal stimulus category x electrode interaction [*F*(4,52) = 3.84, *p* = 0.072]. Separate stimulus category x electrode ANOVAs were thus performed for each pairwise combination of stimulus category (TBR vs new, TBR vs TBF, TBF versus new). The comparison of TBR and new items yielded a main effect of stimulus category [*F*(1,13) = 126.78, *p*<0.001], as did the comparison of TBF and new items [*F*(1,13) = 65.88, *p*<0.001], indicating more positive amplitudes for both TBR and TBF words compared to new words. The stimulus category x electrode interaction was significant for the TBF-new comparisons [*F*(2,26) = 7.55, *p*<0.01] and marginal for the TBR-new comparison [*F*(2,26) = 3.97, *p* = 0.068], in both cases reflecting greater old/new effects at Fz and Cz relative to Fpz. By contrast, the comparison of TBR and TBF words yielded no main effect of stimulus category [*F*(1,13) = 1.08, *p* = 0.318] or stimulus category x electrode interaction [*F*(2,26) = 0.43, *p* = 0.526].

As with the earlier interval, analyses of ERPs over the 450–650 ms window yielded a main effect of stimulus category [*F*(2,26) = 60.84, *p*<0.001] as well as a stimulus category x electrode interaction [*F*(4,52) = 7.26, *p*<0.05]. Focused stimulus category x electrode ANOVAs indicated significant main effects of stimulus category both when TBR words were compared to new words [*F*(1,13) = 85.63, *p*<0.001] and when TBF words were compared to new words [*F*(1,13) = 57.21, *p*<0.001]. For both comparisons, significant stimulus category x electrode interactions [*p’s* <0.05] indicated greater old/new effects at Cz and Pz relative to Oz. Importantly, a significant main effect of stimulus category also emerged when TBR items were compared to TBF items [*F*(1,13) = 11.76, *p*<0.01], indicating more positive ERPs for TBR relative to TBF items. This finding thus confirmed the presence of a directed forgetting effect on the explicit memory test from 450–650 ms. The stimulus category x electrode interaction for this comparison was nonsignificant [*F*(2,26) = 2.33, *p = *0.151].

#### ERPs – Implicit memory test

The ERP waveforms and topographical plots corresponding to the implicit memory test are depicted in Figures 2a and 2b (right panels). As with analyses of the explicit memory test, ERP old/new effects were visible for both TBR words and TBF words compared to new words over both the 350–450 ms and 450–650 ms intervals. However, ERPs during the implicit memory test did not appear to differ between TBR and TBF words over either time interval. Formal statistical analyses over the 350–450 ms and 450–650 ms intervals substantiated these observations.

Over the 350–450 ms interval, a significant main effect of stimulus category emerged [*F*(2,26) = 6.26, *p*<0.05]. However, there was no significant stimulus category x electrode interaction [*F*(4,52) = 0.89, *p* = 0.363]. Separate stimulus category x electrode ANOVAs were performed for each pairwise combination of stimulus category (TBR vs new, TBR vs TBF, TBF versus new). The comparison of TBR and new items yielded a main effect of stimulus category [*F*(1,13) = 8.47, *p*<0.05], as did the comparison of TBF and new items [*F*(1,13) = 15.88, *p*<0.01], indicating more positive amplitudes for both TBR and TBF words compared to new words. However, this effect was nonsignificant for the comparison of TBR and TBF items [*F*(1,13) = 0.50, *p* = 0.49]. No stimulus category x electrode interactions emerged for any comparison [*F*(2,26) = 0.60, *p* = 0.45 for TBR vs new; *F*(2,26) = 2.11, *p* = 0.18 for TBF versus new; *F*(2,26) = 0.23, *p = *0.64 for TBR versus TBF].

As with the earlier interval, analyses of ERPs at 450–650 ms yielded a main effect of stimulus category [*F*(2,26) = 10.27, *p*<0.01]. A significant stimulus category x electrode interaction also emerged [*F*(4,52) = 5.38, *p*<0.05]. Focused stimulus category x electrode ANOVAs indicated significant main effects of stimulus category both when TBR words were compared to new words [*F*(1,13) = 16.64, *p*<0.01] and when TBF words were compared to new words [*F*(1,13) = 14.86, *p*<0.01]. For both comparisons, significant stimulus category x electrode interactions [*p’s* <0.05] indicated greater old/new effects at Cz and Pz relative to Oz. Finally, the comparison of TBR and TBF words yielded neither a main effect of stimulus category [*F*(1,13) = 1.90, *p = *0.191], nor a stimulus category x electrode interaction [*F*(2,26) = 1.31, *p = *0.273].

In summary, the old/new effects from 450–650 ms were larger for TBR than for TBF items on the explicit memory test, during which participants were intentionally attempting to remember items from the study phase, but not on the implicit memory test, when any memory retrieval would presumably have been incidental.

## Discussion

Previous investigations of directed forgetting have focused largely on its effects on later intentional recognition or recall success. By contrast, incidental or involuntary retrieval experiences have received insufficient emphasis in the directed forgetting literature. Whereas some relevant studies have included implicit memory tests [10], [17]–[22], the focus of these studies has been on whether or not DF instructions can influence implicit memory phenomena such as priming. To our knowledge, the present study was the first to identify neural signals of incidental recollection for TBR and TBF items during an implicit memory test, thus allowing for the investigation of DF effects on incidental explicit memory retrieval.

As predicted, we obtained significant P600 old/new effects for both TBR and TBF words in both the explicit memory test (old/new recognition) and the implicit memory task (lexical decision). P600 potentials have been linked in prior research to explicit memory retrieval, and particularly to recollection [29]–[33]. Thus, the present findings join others [7], [24], [26] in providing evidence of robust recollection for studied words during the lexical decision task, despite the fact that performance on this task neither encourages nor requires explicit memory retrieval (e.g., [36]). Most interestingly, analyses of DF effects on P600 potentials revealed differential effects on recollection that occurred during the recognition test (intentional recollection) and recollection that occurred during the lexical decision task (incidental recollection). As in previous studies of directed forgetting (e.g., [21], [22], [34]), P600 potentials on the recognition test were larger for TBR relative to TBF items. By contrast, P600 potentials on the lexical decision task were equal in magnitude for both types of studied items. This result suggests that, at least in the paradigm used here, DF instructions served to reliably influence later intentional but not incidental recollection.

Intentional retrieval is, by definition, a more effortful and controlled process relative to incidental retrieval. For this reason, it may seem counterintuitive to suggest that the former process may be more susceptible than the latter to directed forgetting instructions. We believe that the present results can be explained through the lens of encoding-specificity theory [13], which posits that the likelihood of retrieval success depends on the degree of similarity between the context that is present during encoding and that which is present during retrieval. Importantly, this “context” can include not only the physical environment present at study, but also the participant’s mental set and the types of internal processing that are applied to the studied stimuli. As previously mentioned, most theoretical accounts of directed forgetting attribute DF effects at least partially to the enhanced encoding of TBR relative to TBF cues. That is, participants cease attempting to commit the TBF items to memory once they receive a “Forget” cue, whereas a “Remember” cue prompts additional elaborative processing, resulting in a memory trace that is more richly bound to its context. Because participants taking an intentional memory test are likely to spontaneously employ retrieval strategies that attempt to reinstate aspects of their internal study context [14]–[16], they are more likely to successfully retrieve memories of TBR relative to TBF items. During a lexical decision test, however, such elaborative retrieval strategies are neither necessary nor encouraged, and thus participants’ recollection may be less influenced by the more richly elaborated memory representations that are present for TBR words.

This account bears some resemblance to the “mental context change” theory of directed forgetting that has been applied to list-method studies in which participants are instructed to forget an entire list of already-encoded stimuli before attempting to remember a new list [11]. Studies that have employed the list method typically find that participants who are instructed to forget the List 1 words recall both fewer words from List 1 and more words from List 2 relative to participants who do not receive this instruction. The mental context change account of this phenomenon suggests that the “Forget” instruction prompts participants to actively induce changes in study strategy or other aspects of their mental context, such that they encode List 2 items in a different internal context that is more easily reinstated at the time of test relative to the List 1 context.

To our knowledge, the present study represents the first time that contextual-reinstatement effects have been brought to bear on theories of item-method directed forgetting. Indeed, this connection prompts new hypotheses about when and how item-level DF instructions will be most effective. For example, the present results suggest that any type of change in external or internal context between study and test will tend to unevenly affect memory for TBR and TBF items, because the latter stimuli are less “bound” to the context in which they were originally studied. Directed forgetting instructions may therefore be most effective when participants’ contextual environment is stable between study and test. Moreover, Brinegar, Lehman, and Malmberg (2013) recently provided evidence that *pre-instatement –* the act of imagining the contextual environment in which a future retrieval attempt will occur during initial study – can allow participants to overcome the deleterious effects of context change on a free recall test [37]. Accordingly, it is possible that pre-instatement instructions can also reduce contextual change effects on directed forgetting. These issues constitute a promising area for additional research.

One caveat to the findings presented here is that, despite the large body of research supporting a relationship between P600 potentials and recollection, these ERPs remain an indirect (and likely imperfect) measure of the amount of recollection that occurs in any given circumstance. It is possible that the relationship between P600 amplitude and recollection is not exclusive, such that there are some incidences of recollection that are not captured by these ERPs (e.g., [38]). For this reason, it cannot be assumed that the differential effects of directed forgetting on intentional versus incidental recollection observed here are universal. Nonetheless, the present findings establish for the first time that retrieval intention interacts with the effects of directed forgetting on at least one neural signature of recollection, and further illustrate the utility of ERPs as a tool to measure aspects of memory retrieval that can be problematic to obtain by verbal report.

We are also not suggesting that directed forgetting instructions will never influence P600 amplitudes during indirect memory tests. On the contrary, there is evidence that the mechanisms that lead to directed forgetting effects can vary across experimental circumstances and are not always limited to the enhanced encoding of TBR items [8]–[12], [39], [40]. In list-method experiments, for example, significant directed forgetting effects on recall have been found following incidental learning [39] in which no intentional encoding strategies should have been employed. These and similar findings have been taken as evidence that *active inhibition* – either of the TBF words themselves or of their associated study context – can be instrumental in producing DF effects. Presumably, the successful deployment of inhibitory processes, at least when applied to the TBF words themselves, will render their corresponding memories less accessible by both intentional and incidental retrieval. It would be interesting to test this hypothesis in future research by examining directed forgetting effects on incidental recollection following list-method rather than item-method instructions.

Interestingly, the relative balance of elaborative encoding of TBR words versus the active inhibition of TBF words is also relevant to an adjacent area of controversy involving the extent to which DF instructions can affect implicit or nonconscious expressions of memory such as priming. Priming effects on lexical decision and certain other implicit memory tasks have been shown to be relatively insensitive to manipulations of the depth or amount of encoding afforded to the words at study (e.g., [24], [25], [41], but see [42], [43]). For this reason, it has been argued that pure selective encoding accounts of directed forgetting would be unable to account for findings of greater priming effects for TBR than TBF words, and that such findings would be more consistent with theories that posit a combination of enhanced encoding of TBR words and active inhibition of TBF words [10]. In the present study, however, directed forgetting did not influence behavior on the lexical decision task. Rather, TBF and TBR words showed equal amounts of priming on this task, as evident in speeded reaction times for both word categories when compared to new words. Likewise, N400 potentials, an ERP index of priming on lexical decision tasks [44], did not differ between TBF and TBR items on either test type.

These results are consistent with a number of prior studies (e.g., [20]–[22]) in which the effects of directed forgetting were limited to explicit memory tasks, and thus which have been taken to support selective-encoding accounts of item-level directed forgetting effects. However, it remains an open question why item-level directed forgetting effects have sometimes been reported on lexical decision or stem completion tests [10], [17], [18], including in studies that used block lengths that were comparable to ours. One potentially relevant difference between the current study and these prior studies is that each of the previous studies utilized only one study-test block per participant. As a result, the experimenters were able to mislead participants into believing that only TBR items would appear on the later memory tests. By contrast, it was necessary for us to incorporate multiple study-test blocks in order to keep the study blocks short while still amassing enough trials for ERP analyses. Thus, our participants were made aware that the memory tests contained both TBR and TBF words. Importantly, this awareness did not prompt our participants to disregard the directed forgetting instructions. Both behavioral and electrophysiological evidence of directed forgetting was found on the explicit memory test. However, it is possible that participants’ knowledge of the test phases caused them to shift their strategy away from attempting to inhibit TBF words, and towards a reliance on enhanced encoding of TBR words.

In future studies, it may be informative to put this speculation to the test by contrasting directed forgetting effects on explicit and implicit memory tests under circumstances that offer more or less of an incentive for TBF words to be inhibited. For example, in a recent study by Cheng et al. [45], participants were misinformed during the study phase (but not during the test phase) that there would be a monetary penalty for every TBF item they later recalled, and that this penalty would exceed the reward offered for remembering a TBR item. ERP data in this study showed evidence of a greater depletion of cognitive resources following TBF relative to TBR words, suggesting that participants attempted to actively inhibit TBR words. It may be fruitful in subsequent research to examine whether directed forgetting effects on both priming and incidental recollection occur differently on lexical decision tasks under these circumstances relative to circumstances in which it is the memory for TBR words that is linked to the highest reward value. This and other subsequent research may offer a more complete picture of mechanisms and consequences of directed forgetting instructions across a wider range of situations.

As previously stated, an important and novel finding of this study is that retrieval intention can interact with encoding intention under some circumstances, such that differences between TBR and TBF items are more evident when retrieval is intentional relative to when retrieval is incidental. This distinction may be particularly relevant when considering possible clinical applications of directed forgetting, such as the potential to reduce negative memory intrusions in conditions such as posttraumatic stress disorder and depression (e.g., [2]). The unwanted memory intrusions that occur in these conditions arguably can be characterized as incidental or unintentional in nature, and yet incidental retrieval has been relatively ignored in directed forgetting research thus far. Our study is the first to show that, in fact, an effect of directed forgetting instructions on intentional retrieval need not imply an effect on incidental retrieval, suggesting that incidental retrieval should play a larger role in directed forgetting research going forward. Covert measures of retrieval such as ERPs are likely to be particularly important in this regard, as such measures offer insight into incidental forms of memory that are particularly difficult to quantify via subjective reports.

## Supporting Information

Figure S1 **ERP differences between TBR and TBF cues during the study phase.** Grey vertical lines mark the 250–450 ms time window. Topographical plots depict the difference between TBR and TBF words during this window.
(TIF)

Text S1 **Analyses of study-phase ERP data.**
(DOCX)
