# Supplementary figures and images for: Retrieval Intention Modulates the Effects of Directed Forgetting Instructions on Recollection
Source: PLoS One. 2014 Aug 20;9(8):e104701. doi: 10.1371/journal.pone.0104701 (PMC4139323; doi:10.1371/journal.pone.0104701)

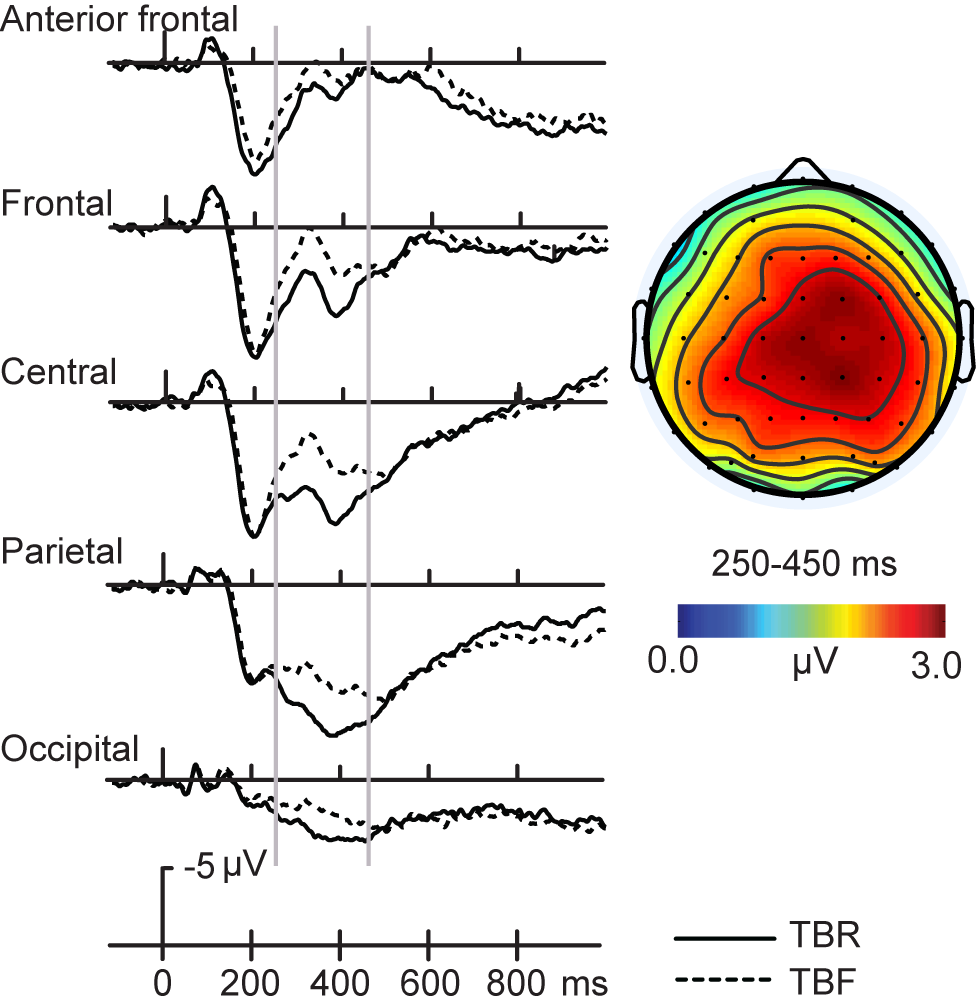

Supplement: Figure S1 — ERP differences between TBR and TBF cues during the study phase. Grey vertical lines mark the 250–450 ms time window. Topographical plots depict the difference between TBR and TBF words during this window. (TIF) [file pone.0104701.s001.tif]
