# Supplementary material for: Retrieval Intention Modulates the Effects of Directed Forgetting Instructions on Recollection
Source: PLoS One. 2014 Aug 20;9(8):e104701. doi: 10.1371/journal.pone.0104701 (PMC4139323; doi:10.1371/journal.pone.0104701)
Supplement: Text S1 — Analyses of study-phase ERP data. (DOCX) [file pone.0104701.s002.docx]

**Supporting Information. Text S1**

**Analyses of study-phase ERP data**

In analyses of study phase data, ERPs were quantified as the average amplitudes for all TBR or TBF stimuli from 250-450 ms after the onset of the directed forgetting cues (the “RR” or “FF” instructions). A 2 (stimulus category: TBF/TBR) x 5 (electrode: Fpz/Fz/Cz/Pz/Oz) ANOVA was conducted for the 250-450 ms window. The results revealed a significant main effect of stimulus category [*F*(1,13) = 20.07, *p* < 0.01] as well as a significant interaction between stimulus category and electrode [*F*(4,52) = 5.20, *p* < 0.05]. Follow-up pairwise comparisons between mean amplitudes to TBR and TBF cues revealed that TBR cues elicited significantly more positive amplitudes at each electrode [*F*(1,13) = 18.61, *p* < 0.01 at Fpz; *F*(1,13) = 21.54, *p* < 0.01 at Fz; *F*(1,13) = 18.27, *p* < 0.01 at Cz; *F*(1,13) = 12.49, *p* < 0.01 at Pz; *F*(1,13) = 9.42, *p* < 0.05 at Oz]. In light of previous findings of greater positive-going ERPs for TBF relative to TBR words around 500 ms (e.g., [[45](#_ENREF_41)]), the above ANOVA was also conducted for the 450-550 ms window. No significant results emerged over this window for either the main effect of stimulus category [*F*(1,13) = 0.40, *p* = 0.544] or the interaction with electrode [*F*(4,52) = 0.51, *p* = 0.491].
